# Identifying species threatened with local extinction in tropical reef fisheries using historical reconstruction of species occurrence

**DOI:** 10.1371/journal.pone.0211224

**Published:** 2019-02-13

**Authors:** Sarah M. Buckley, Tim R. McClanahan, Eréndira M. Quintana Morales, Victor Mwakha, Jatieno Nyanapah, Levy M. Otwoma, John M. Pandolfi

**Affiliations:** 1 Australia Research Council Centre of Excellence for Coral Reef Studies, University of Queensland, Brisbane, Queensland, Australia; 2 Wildlife Conservation Society, Marine Programs, Bronx, New York, United States of America; 3 The Pennsylvania State University, University Park, Pennsylvania, United States of America; 4 Kenya Marine and Fisheries Research Institute, Mombasa, Kenya; 5 Leibniz Centre for Tropical Marine Research, Bremen, Germany; Department of Agriculture and Water Resources, AUSTRALIA

## Abstract

Identifying the species that are at risk of local extinction in highly diverse ecosystems is a big challenge for conservation science. Assessments of species status are costly and difficult to implement in developing countries with diverse ecosystems due to a lack of species-specific surveys, species-specific data, and other resources. Numerous techniques are devised to determine the threat status of species based on the availability of data and budgetary limits. On this basis, we developed a framework that compared occurrence data of historically exploited reef species in Kenya from existing disparate data sources. Occurrence data from archaeological remains (750-1500CE) was compared with occurrence data of these species catch assessments, and underwater surveys (1991-2014CE). This comparison indicated that only 67 species were exploited over a 750 year period, 750-1500CE, whereas 185 species were landed between 1995 and 2014CE. The first step of our framework identified 23 reef species as threatened with local extinction. The second step of the framework further evaluated the possibility of local extinction with Bayesian extinction analyses using occurrence data from naturalists’ species list with the existing occurrence data sources. The Bayesian extinction analysis reduced the number of reef species threatened with local extinction from 23 to 15. We compared our findings with three methods used for assessing extinction risk. Commonly used extinction risk methods varied in their ability to identify reef species that we identified as threatened with local extinction by our comparative and Bayesian method. For example, 12 of the 15 threatened species that we identified using our framework were listed as either least concern, unevaluated, or data deficient in the International Union for the Conservation of Nature red list. Piscivores and macro-invertivores were the only functional groups found to be locally extinct. Comparing occurrence data from disparate sources revealed a large number of historically exploited reef species that are possibly locally extinct. Our framework addressed biases such as uncertainty in priors, sightings and survey effort, when estimating the probability of local extinction. Our inexpensive method showed the value and potential for disparate data to fill knowledge gaps that exist in species extinction assessments.

## Introduction

While global extinctions are rare, local extinctions at population or smaller–scale geographies are becoming increasingly common [1,2]. However, due to limited funding for broad and intensive sampling [3] and the need for robust statistics to estimate local extinction [4,5], identifying at-risk species remains challenging. Being able to identify species at risk of extinction is important, as even local extinctions have implications for the food diversity and security of dependent communities [6,7] and ecosystem functional diversity, productivity, and resilience [8–10]. In particular, the loss of key predators can fundamentally change fisheries ecosystems and associated services [11–13]. Consequently, there is a need for rapid and inexpensive methods to assess the vulnerability of species to extinction and to assess lost diversity and ecological services [5,14].

Vulnerability to extinction is not random [15] and factors such as geographical range size, evolutionary history, life history traits, and marketability can determine susceptibility to extinction [16–18]. Biological traits such as a large body size, late sexual maturity, and long generation time are considered among the most consistent and reliable correlates of extinction vulnerability in marine fish [19–21]. Moreover, carnivorous fish are most sensitive to fishing pressure and extinction because of traits that increase their vulnerability to extinction [16, 22–27]. The expected vulnerabilities of carnivores provoked research that has identified historically-exploited species that are now locally extinct. For example, Ferretti et al. [28] reconstructed the occurrence of two locally extinct, commercially important sawfish species (*Pristis pristis* and *P*. *pectinata*) from 1576 to the present using historical documents, images, museum specimens, and catch records. They demonstrated that these species went extinct in the Mediterranean between the 1960s and 1970s. Similarly, Drew et al. [29] compared shark teeth records from a museum repository with contemporary species lists, revealing the local extinction of spot tail (*Carcharhinus sorrah*) and dusky (*C*. *obscurus*) sharks from Kiribati (Oceania) prior to the onset of scientific monitoring. Both studies used a range of historical and contemporary data to reconstruct the occurrence of species over time in order to accurately estimate the rate of decline and make conclusions about local extinction. They show local historical data are potentially well suited to document past range contractions, declines in abundance, and extinctions [30, 31].

Varied extinction risk methods have been introduced to assess the vulnerability of species to extinction [25–27]. Comparing established extinction risk methods with approaches that make use of both historical and modern data sources can potentially highlight the efficacy of existing extinction risk methods. Nevertheless, while the comparison of historical and modern records can be useful, it can also be challenged by data limitations. Sources can differ in their time-span, location, precision in timing, geographic area, biases in observing and identifying species occurrences, and other method and data-inherent uncertainties [32–35]. For example, traditional ecological knowledge is often used to reconstruct species occurrence and abundance histories, but suffers from bias in memories and inaccuracies in reporting of historically-exploited species abundances [35]. Additionally, archaeological records are influenced by a number of factors that include variable preservation rates, biases in sampling and excavation methods, and changing fishing and discard behaviors [31]. To better account for these biases, recent Bayesian extinction analyses have estimated the probabilities of survey effort bias and false positives [36,37].

Historical and contemporary species occurrence studies have generally been confined to regions with historical written documents and quantitative records [16]. In contrast, developing countries that rely heavily on natural resources and fishing often have few written baseline records. Therefore, extinction risk methods remains a practical approach to predict extinction risk among coral reef fishes [22, 23]. In countries without long written traditions, archaeological records are another alternative to detecting species occurrences [30, 31,38]. Here we investigated historically-exploited coral reef fish species in Kenya to determine taxa likely to be threatened by local extinction. Our central hypotheses were: (i) that carnivores would be among the most frequently threatened species and (ii) that our historical analysis method would corroborate the results of other well established extinction risk methods. These hypotheses were based on the premise that population declines are non-random and therefore similarly detectable by disparate methods [16,17,26,39]. We extracted species sightings data from the disparate data sources, archaeological remains, naturalists’ species lists, catch assessment records, and underwater surveys to reconstruct changes in the presence of exploited fish. First, we reconstructed occurrences of each historically-exploited species over historical time from all available sources of data. Second, we showed which historically exploited reef species were absent from the contemporary records. Third, we applied Bayesian extinction likelihood analyses to account for differences in our data collection methods and types [36]. Finally, species that we identified as threatened with local extinction were compared with the results of three established extinction risk methods: vulnerability to extinction, vulnerability to fishing, and functional vulnerability.

## Materials and methods

Kenya provides a unique opportunity to investigate a variety of detection methods because of a mixture of data sources including written, archeological, contemporary catch, and underwater visual census records [38, 40]. Modern Kenyan artisanal fishers exploit over two hundred and seventy coral reef fish species, yet the status of the majority of these species remains unknown. Kenya’s coastal artisanal fisheries date back to before the 8th century [41] and are a central part of the subsistence, culture, and economy of coastal people [41,42]. Over the past few decades, Kenya’s coral reef fisheries have experienced high fishing pressure [43]. This has raised concerns that fisheries are overexploited, as reflected by a decrease in biomass and yields, particularly top predators, such as groupers [38,44]. In contrast, the historical catch assemblage (750–1350 Common Era (CE)) was composed of fish species with larger mean body size and slower growth and mortality rates than taxa common in the modern assemblage (1990–2010) [38]. Fishers and managers are therefore concerned that local extinction of the most vulnerable taxa may be occurring.

### Species sightings data

**Fig 1:**
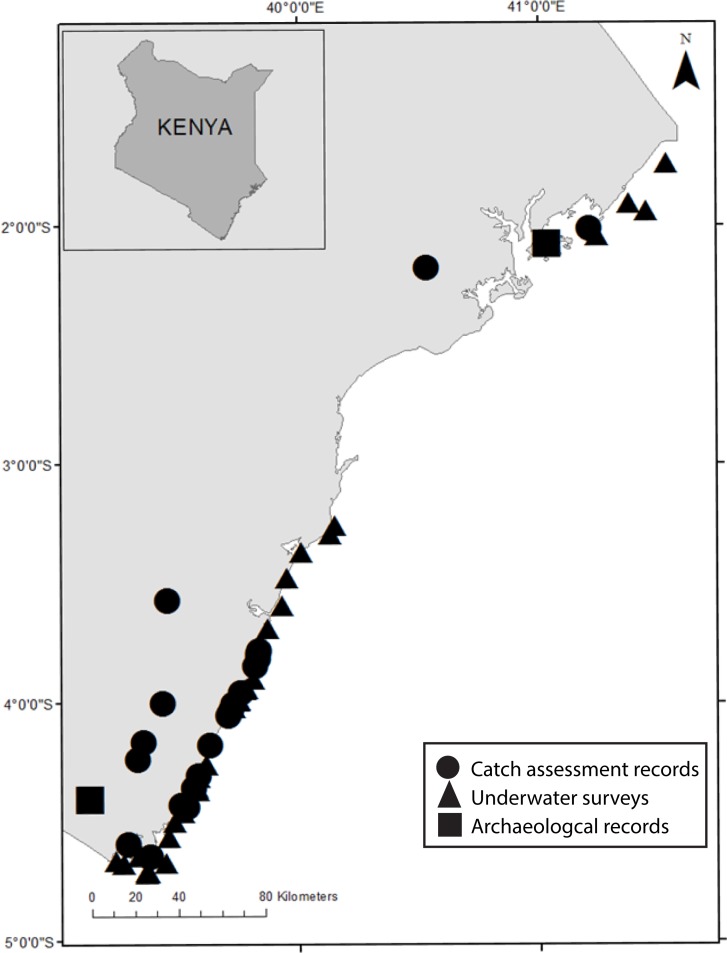
Map of Kenya showing the locations of various data sources for reef fish species occurrences through time.

Kenya has among the highest quality species sightings and catch trend data known for a developing country. Further, Kenyan reef fish species occur throughout much of the Indian Ocean and coral reefs worldwide. We defined reef fish species as shallow-living (<100 m depth), tropical/ subtropical, benthic or benthopelagic fishes that either associate with hard bottom substrates composed of coral, algae, or dead coral reef, or alternatively, that occupy adjacent sandy substrate and seagrass. We reconstructed occurrences of each historically-exploited species using four data sources. These included archaeological records (750–1500), naturalists’ species lists (1759–2003), catch assessment surveys (1995–2013), and underwater surveys (1991–2013). The archaeological records were only from two sites along the Kenyan coast in the extreme north and south, whereas the catch assessments and underwater surveys were conducted more broadly (Fig 1). The spatial resolution of the naturalist’s species lists is unknown.

#### Archaeological records

Archaeological data comprise reported fish remains found in published or publicly available sources. This included two archaeological sites along the Kenyan coastline: Shanga (750–1400 CE) [45, 46] in the northern region and Vumba Kuu (1300–1500 CE) [41] in the southern region (Fig 1). Excavations conducted at Vumba Kuu and Shanga under research permits obtained from the Kenya Ministry of Science and Technology and the Vice President’s Office and the National Museums of Kenya respectively. Both the Shanga and Vumba Kuu fish remains assemblages were analyzed using standard zooarchaeological methods [47,48], aided by morphological comparison with modern fish skeleton collections at the National Museums of Kenya. We collated fish species abundance data according to the human settlement phases to determine changes between 750 and 1500 CE. At Shanga, distinct stratigraphic layers represented sequential building construction phases of approximately 50 years in length (i.e. 750-800CE). These phases outline the development of Shanga from the 8th to 15th century, a period of growing coastal urbanization and trade at both local and regional scales. At the height of this period, people inhabited Vumba Kuu for approximately 200 years. Excavations at Vumba Kuu did not yield evidence of distinct phases of occupation. We recorded the presence of each species in each phase that ranged from approximately 50 to 200 years (i.e. 750–800 CE). The final year of the settlement phase was used to represent the last year a species was observed.

#### Naturalist’s species lists

Naturalist’s species lists were derived from the Kenyan office of the Wildlife Conservation Society (WCS) and the published literature. We conducted standardized searches using keywords such as scientific and common names for species, as well as any relevant name change (S1 Table). Naturalist’s compiled these species lists from species sightings, species occurrence lists, photographs and museum specimens between 1759 and 2003. From these records, we extracted information on the presence of each historically-exploited species and the recorded year. We verified species classification and occurrences with online biodiversity databases, including World Register of Marine Species and FishBase [49].

#### Catch assessment records

Catch assessment records were acquired from WCS-Kenya, which has collected data on landed fish from 25 landing sites since 1995 (Fig 1). Fishing is typically conducted from the shore to the outer reef in sand, coral, and seagrass habitats of the fringing reef lagoon [40]. WCS-Kenya catch records classified 185 landed fish to the species level. The landing site, date, gear used, number of individuals per species, and standard lengths were recorded [50, 51]. A total of 278 days and 25 locations were sampled over the 18-year period. These data were extracted and used to aid in construction of species occurrence during the contemporary period. From these records, we extracted information on the presence of each historically-exploited species (species that were exploited between 750-1500CE) and the recorded year.

#### Underwater survey records

Our final resource was underwater surveys from WCS-Kenya conducted between 1991 and 2012 at 44 sites along the Kenyan fringing reef (Fig 1). Surveys were conducted in fisheries closures and fished reefs with various types of gear management. In each site, a single investigator recorded fish to species level in replicate 500m^2^ belt transects [52]. A total of a 199 fish species in selected families were observed in 404 transects (S2 Table). We extracted all occurrence information specific to our historically-exploited species list including the species, date, and location. These observations were then used to construct species occurrence during the contemporary period. From these records, we extracted information on the presence of each historically-exploited species and the recorded year.

### Data limitations

A number of biases and limitations exist when reconstructing species occurrence from disparate data sources, including differences in types of sighting and the reliability of the sighting. For example, the archaeological remains represent only captured species and not the full assemblage of fish. Species may have existed in the past but were not captured and therefore undetected and impossible to assess for extinction risk. Variable levels of preservation and sampling strategies are also affect occurrences in the archaeological record. Other limitations include variation in the temporal resolution and data gaps, primarily in our study from 1500–1991 CE. Unlike archaeological data, species identified in naturalists’ lists provide a single date when a species was observed. Spatial scale and resolution is also variable and the archaeological data here represents two distinct regions, whereas catch assessments and underwater surveys are distributed more broadly but differ in their coverage of habitats and the species assemblages. These limitations can be mitigated by the Bayesian extinction analysis explained below.

### A framework for identifying species vulnerable to local extinction

We used a cohesive framework that included two steps. The first step compares across data sources to identify which reef species are absent or rare in current records compared to the past. The second step is to leverage across data sources to determine the probability that those species identified in step 1 may be locally extinct while accounting for uncertainties in disparate data sources.

#### (1) Identifying reef species absent from contemporary records

We compiled a list of reef species that were observed in the archaeological record but were absent or rare in the contemporary data sets (catch assessment records and underwater surveys). Initially, every species was examined but the list was subsequently refined to target and by-catch reef fish species. We also considered reef species found in the archaeological record that were rare in the catch assessment records and underwater surveys, i.e. since 1991, and included these in the list of missing reef species. A species was considered rare if it made up <1% of the catch assessments (<15 fish observed in total landings) or <1% of the biomass recorded in underwater surveys (<2 fish observed in total surveys) [31]. Historically exploited reef fish were considered as ‘absent’ when they were present in archaeological records but absent or rare in the naturalist’s species lists, catch assessment records and underwater surveys. If species were categorised as ‘absent’, this signified that the species were potentially threatened with local extinction.

#### (2) Estimating the probability of local extinction

We used the Bayesian extinction analysis to verify if our framework identified historically exploited species absent from modern data sources as species that show a high probability of local extinction. Bayesian extinction analysis addresses whether a species is rarely sighted, or is actually locally extinct. It does this by computing the probability of a species being extant using sightings data from disparate data sources (S1 Datasheet) [36]. Following the approach derived by Lee [36], the analysis accounted for the small sample size, survey effort, and the uncertainty of sightings in different types of data sources. To implement the Bayesian model, we derived estimates for the priors (P(*XT*), *P*(*E*)) and uncertainty around the priors, for detectability for definite sightings (*d*_1_), detectability for uncertain (*d*_2_) sightings, false detectability for uncertain sightings (*f*_2_) and survey effort (*s*). We present results for the grouper *Epinephelus coioides* to describe the approach, which infers extinction from sighting records [36].

Two priors were derived for this method: the prior probability of seeing the species, given it is extant given the sighting record P(*XT*) and a prior belief that the species went extinct in a particular year after a sighting *P*(*E*) [36].

$$P(X)=\left[\frac{T_{N}}{T}‑\delta ,\frac{T_{N}}{T}+\delta \right]$$

$$P(E)=\left[\frac{1}{T}‑\delta ,\frac{1}{T}+\delta \right]$$

**Fig 2:**
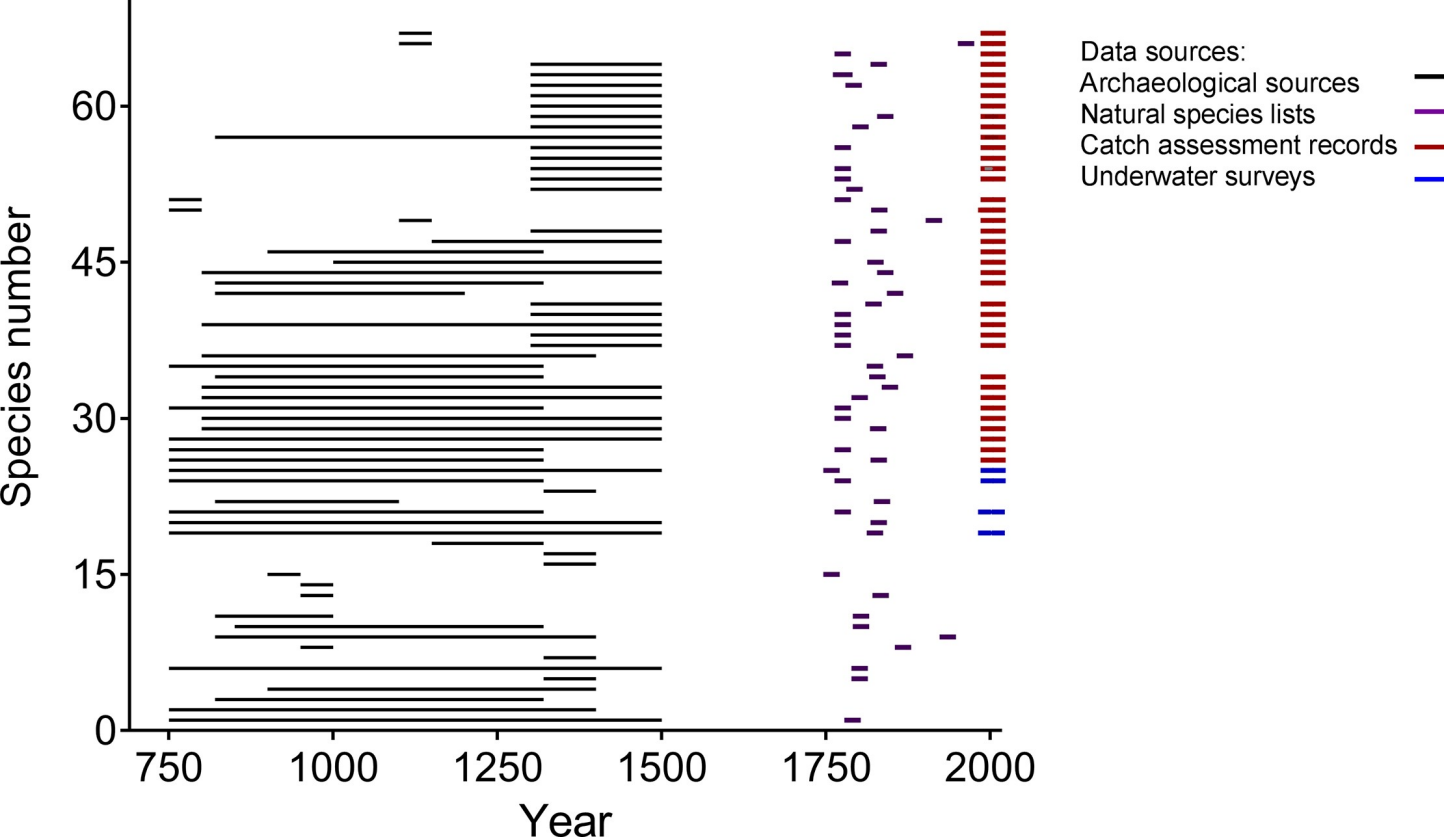
Sightings of historically exploited species reconstructed over time using a suite of disparate data sources. The colour of the line represents from where the coral reef species data were sourced from: archaeological remains (black), naturalists species lists (purple), catch assessment records (red) and underwater surveys (blue).

*T*_*N*_ is the number of years from the first to the last sighting and *T* is the number of years from the first sighting to the year when the extant estimate was made. For each species, we extracted sightings data from the archaeological remains, naturalist’s species lists, catch assessments records, and underwater surveys to reconstruct a time series of sightings (Fig 2). When the specific year was not available, we used the last year of the time period when the occurrence was noted. The equations allow for uncertainty around the prior so the estimate lies in the center of each range where δ = 0.1. For *E*. *coioides*, we would have

$$P(X)=\left[\frac{T_{N}}{T}-\delta ,\frac{T_{N}}{T}+\delta \right]=\left[\frac{200}{720}-0.1,\frac{200}{720}+0.1\right]=[0.180,0.38]$$

and

$$P(E)=\left[\frac{1}{T}‑\delta ,\frac{1}{T}+\delta \right]=\left[\frac{1}{720}‑0.1,\frac{1}{720}+0.1\right]=[‑0.098,0.101]$$

Detectability is for definite sightings (*d*_1_) and was based on the number of definite sightings (*S*) that occurred during the time that the species was known to be extant (*T*_*N)*_. Definite sightings are fish bones from archaeological records.

$$d_{1}\approx \frac{S}{T_{N}}$$

In this case of *E*. *coioides*, at least one set of fish bones was obtained in 1300 and 1500. For the *E*. *coioides*, the detectability for specimens (*d*_*1*_) is

$$d_{1}\approx \frac{S}{T_{N}}=\frac{2}{200}=0.01$$

There is approximately 0.01 probability of obtaining a specimen of *E*. *coioides*, if it is extant, since the specimen was only recorded twice in the 200 years it was known to be extant. To account for uncertainty in the estimate, detectability (*d*_*1*_) lies in the center of the chosen range, that *d*_1_ = [0,0.02].

Detectability for uncertain sightings (*d*_2_) was based on the probability of uncertain sightings being true.

$$d_{2}=[d\;x\;S_{U},d\;x\;S_{L}]=dS_{U},dS_{L}$$

There were three uncertain sightings in 1882, 1996 and 2012. For this step of the analysis we assessed the uncertainty of sighting a species based on the sighting type. To achieve this, we used Birdlife International scoring criteria that have been used on a range of taxa including mammals, fish, and birds [36, 53–55]. The scoring criteria for the various types of uncertainty of each sighting were based on the quality of evidence and circumstance of the record as follows: (1) catch (0.90–0.95 reliability) and (2) underwater surveys and naturalist’s species list (0.80–0.90 reliability).

The 1882 uncertain sighting record is from the naturalist’s species list and has a sighting probability of 0.80–0.90 of being true. We considered the upper and lower limits, S_U_ and S_L_ respectively, of the probability of the sighting being true_._ Thereby, for every ten sightings of the *E*. *coioides*, which was only found in the catch assessment records, eight are considered true. For the *E*. *coioides* the detectability for uncertain sightings (*d*_*2*_) is

$$d_{2}=[d\;x\;S_{U},d\;x\;S_{L}]=dS_{U},dS_{L}=[0.01\;x\;8,0.01\;x\;9]=[0.08,0.09]$$

The 1996 and 2012 uncertain sighting records are from the catch assessment records and have a sighting probability of 0.90–0.95 of being true. We considered the upper and lower limits, S_U_ and S_L_ respectively, of the probability of the sighting being true_._ Thereby, for every ten sightings of the *E*. *coioides*, which was only found in the catch assessment records, nine are considered true. For the *E*. *coioides* the detectability for uncertain sightings (*d*_*2*_) is

$$d_{2}=[d\;x\;S_{U},d\;x\;S_{L}]=dS_{U},dS_{L}=[0.01\;x\;9,0.01\;x\;9.5]=[0.09,0.095]$$

The false detectability for uncertain sightings (*f*_2_) was based on the probability of uncertain sightings being false.

$$f_{2}=[dS_{U}‑d,\;dS_{L}‑d]=fS_{U},fS_{L}$$

We derived the false detectabilities for the 1882 uncertain sighting record (*f*_2_). Again, we considered the upper and lower limits, S_U_ and S_L_ respectively, of the probability of the sighting being true and as before, for the lower limit for every ten sightings, eight are true. In which case, after extinction one would expect the remaining two sightings to be false, that is *f*_2_ = *d*_2_ –*d*_*1*_. For *E*. *coioides*, the false detectability for uncertain sightings (*f*_*2*_) is

$$f_{2}=[dS_{U}‑d,dS_{L}‑d]=fS_{U},fS_{L}=[0.08–0.01,0.09‑0.01]=0.07,0.08$$

We derived the false detectabilities for the 1996 and 2012 uncertain sighting records (*f*_2)_. Again, we considered the upper and lower limits, S_U_ and S_L_ respectively, of the probability of the sighting being true_._ and as before, for the lower limit for every ten sightings, nine are true. In which case, after extinction one would expect the remaining sighting to be false, that is *f*_2_ = *d*_2_ –*d*_*1*_. For *E*. *coioides*, the false detectability for uncertain sightings (*f*_*2*_) is

$$f_{2}=[dS_{U}‑d,dS_{L}‑d]=fS_{U},fS_{L}=[0.090–0.01,0.095‑0.01]=0.080,0.085$$

We then assessed the likelihood of sighting a species based on the survey effort (*s*). Survey effort (*s*) accounted for both survey detectability (*d*_*s1*_, *d*_*s2*_), i.e. the probability of a sighting during a survey given that the species is extant, and survey false detectability (*f*_*s1*_, *f*_*s2*_), i.e. the probability of a false sighting during a survey given that the species is extinct. The likelihood of sighting a species based on the survey effort was as follows: (1) for catch assessment, a 0.70–0.90 detectability (*d*_*s1*_, *d*_*s2*_) if extant and 0–0.70 false detectability if extinct (*f*_*s1*_, *f*_*s2*_), (2) for naturalist’s species lists, a 0.40–0.60 detectability if extant (*d*_*s1*_, *d*_*s2*_) and 0–0.40 false detectability if extinct (*f*_*s1*_, *f*_*s2*_), and (3) for underwater surveys, 0.60–0.80 detectability if extant (*d*_*s1*_, *d*_*s2*_) and 0–0.60 false detectability if extinct (*f*_*s1*_, *f*_*s2*_). Survey effort values are based on the extent of the species occurring, quality of observers and the frequency, temporal and spatial coverage of the survey (S1 Fig). In this study three of the data sources could be quantified by survey effort.

We applied this method to obtain the probability that species evaluated under our framework as threatened with local extinction were, in fact, still extant in 2015. We also applied this method to those species we considered extant but too rare to obtain a probability that they were extant today. The year 2020 was used as the year of analysis, as the model failed in cases where we sought the probability that a species was extant in 2015 when the sighting input was 2015. We entered our range of estimates into Lee’s [36] numerical tool. We showed the implementation of the *E*. *coioides* example in a screenshot of the Excel spreadsheet (S2 Fig). The example shows the inputs and outputs of the model and how the p-value was determined. The result is a p-value, a statistically assigned probability that a species is still extant, given the observed data. The lower the p-value, the less likely a species is still extant.

### Comparing extinction risk methods

We compared the results of our framework to identify fish species threatened with local extinction with those of three other established extinction risk methods. The extinction risk methods were functional vulnerability [56], vulnerability to fishing [23], and the International Union for the Conservation of Nature (IUCN) Red List status.

Functional vulnerability is the decrease in functional diversity following the local extinction of several species from the same functional group [57]. To assess functional vulnerability we classified species into functional groups defined in Wilson et al. [56], using existing literature and Fish Base [49]. Functional groups directly differentiate among groups of species based on trophic guilds, dietary specialization and life history characteristics. We used the number of species threatened with local extinction per group as a simple surrogate for functional vulnerability.

Vulnerability to fishing is the assessment of the intrinsic extinction vulnerabilities of a species to fisheries exploitation. To assess vulnerability to fishing, we classified species into vulnerable groups as defined by Cheung [23] using the existing literature, Fish Base [49] and expert knowledge. Vulnerability to fishing uses a fuzzy logic expert system that takes into account eight life-history and ecological characteristics (body size, longevity, age at maturity, von Bertalanffy growth parameter, natural mortality, fecundity, spatial behavior, geographic range) that makes species vulnerable to fisheries exploitation. Species were placed into categorical groups based on level of vulnerability to fishing from the estimate produced from the fuzzy logic system [23].

The IUCN red list assessment is a global assessment of a species risk of extinction and is widely accepted as the definitive index. The IUCN approach is to assess a species’ risk to extinction using five quantitative criteria to establish the Red List of Threatened Species [58]. These criteria are used to classify species into different levels of extinction risk such as endangered, vulnerable or least concern [58]. Species are assigned a status or category of extinction risk based is based on a combination of extinction theory, threatening processes and species differences [59]. All Red List status data are from the IUCN Red list website.

We classified the species our analysis found as threatened with local extinction into their respective functional group, vulnerability to fishing groups and IUCN Red List status using data available in FishBase [49] and IUCN Red list. Each respective extinction risk method was used to determine a level of extinction risk for each species. We compared our prediction for the species identified as potentially locally extinct from the Bayesian extinction analysis with the extinction risk assigned under the three other extinction risk methods.

## Results

Our framework was used to test the first hypothesis which proposed that carnivores would be the group most frequently threatened with local extinction. The first step of the framework, comparison of occurrence data from archaeological records with occurrence data from contemporary records, showed that a total of 67 fish species were exploited between 750 and 1500 CE, whereas 185 fish species were exploited between 1995 and 2014 CE. Our absence/presence assessment identified a total of 23 out of these 67 species to be ‘absent’ in the catch assessment and underwater surveys from 1991 onwards (Fig 2, Table 1, S3 Table). Of the 23 threatened species, 9 were rare and 14 were absent from the contemporary records (catch assessment records and underwater surveys) and considered threatened with local extinction. The risk of local extinction was not uniform and species in the family Serranidae showed the highest frequency (n = 7) followed by Haemulidae (n = 5). Carangidae and Sparidae had three and two missing species, respectively, while the Lamnidae, Lutjanidae, Plotosidae, Scaridae, and Stegostomatidae each had one missing species (Table 1).

**Table 1:** List of species potentially threatened with local extinction based on historically-exploited species that were ‘absent’ in the modern data sources (catch assessment records, underwater surveys). The probability that the at-risk species identified were locally extinct. Bayesian extinction analyses are based on specimens from the archaeological record and catch records, and sightings from underwater surveys and naturalist’s species lists. Species (with functional group) are listed in order of most likely to be locally extinct. The P-values are derived from the Bayesian extinction analysis and represents the likelihood that a species is extant today. The lower the P-value, the less likely the species is extant.

Species	Family	Functional group	P-value
*Alectis indica*	Carangidae	Piscivore	<0.0001*
*Cephalopholis aurantia*	Serranidae	Piscivore—Macro-invertivore	<0.0001*
*Epinephelus coeruleopunctatus*	Serranidae	Piscivore	<0.0001*
*Epinephelus lanceolatus*	Serranidae	Piscivore	<0.0001*
*Isurus paucus*	Lamnidae	Piscivore	<0.0001*
*Plectropomus punctatus*	Serranidae	Piscivore	<0.0001*
*Plotosus limbatus*	Plotosidae	Piscivore	<0.0001*
*Dermatolepis striolata*	Serranidae	Piscivore	0.0001*
*Plectorhinchus plagiodesmus*	Haemulidae	Piscivore	0.0001*
*Plectorhinchus sordidus*	Haemulidae	Piscivore	0.0001*
*Stegostoma fasciatum*	Stegostomatidae	Piscivore—Macro-invertivore	0.0008*
*Acanthopagrus berda*	Sparidae	Macro-invertivore	0.0010*
*Lutjanus argentimaculatus*	Lutjanidae	Macro-invertivore	0.0010*
*Atule mate*	Carangidae	Macro-invertivore	0.0014*
*Rhabdosargus sarba*	Sparidae	Piscivore—Macro-invertivore	0.0030*
*Epinephelus coioides*	Serranidae	Piscivore	0.3112
*Scarus russelii*	Scarine Labridae	Detritivore	0.3150
*Diagramma pictum*	Haemulidae	Piscivore—Macro-invertivore	0.3162
*Selar crumenophthalmus*	Carangidae	Piscivore—Macro-invertivore	0.3179
*Epinephelus malabaricus*	Serranidae	Piscivore	0.4033
*Lethrinus enigmaticus*	Lethrinidae	Macro-invertivore	0.4096
*Pomadasys argenteus*	Haemulidae	Macro-invertivore	0.4119
*Pomadasys maculatus*	Haemulidae	Piscivore—Macro-invertivore	0.4168

P-values asterisked (*) are significant at <0.05 level.

The second step of the framework, the Bayesian extinction analysis, tested the likelihood that the 23 species identified as locally extinct by the first step of the framework are extant today. The model accounted for the uncertainties in detectability and survey effort throughout the disparate data sources. The Bayesian extinction model reduced the total number of species likely to be locally extinct, from 23 to 15 species. The 15 species are possibly locally extinct because they showed a very low probability of being extant today, >1% (Table 1). All of these species were carnivores, either piscivores or macro-invertivores. For the eight species determined as extant, but rare in the contemporary record, the probabilities of being extant in 2020 ranged between 0.31 and 0.41. Interestingly seven out of the eight species the framework identified as threatened with local extinction but the model designated as extant were carnivores too.

Finally, species’ functional vulnerability was the only extinction risk method that corroborated those species our framework identified as threatened with local extinction. The species threatened with local extinction occupied only two functional groups, piscivores and macro-invertivores. Nine species of piscivores, three species of macro-invertivores, and six species associated with both categories were identified as threatened with local extinction (Table 2). Vulnerability to fishing was a moderate method in verifying the species our framework identified as locally extinct. The method classified nine of the 15 species as high to very high vulnerability to fishing (Table 2). The IUCN red list listed only three species as high risk to extinction that our framework identified as locally extinct. The IUCN Red List categorised one of the locally extinct species, namely *Stegastoma fasciatum*, as endangered and two of the locally extinct species, namely *Epinephelus lanceolatus* and *Isurus paucus* as vulnerable. Five of the fifteen species were not assessed, four species were listed as Least Concern, and three species were Data Deficient (Table 2).

**Table 2:** Comparison of species identified as possibly locally extinct by our framework with three established extinction risk methods. Our two step framework identified species threatened with local extinction. Step one identified reef species absent from contemporary records and step two estimated the probability of local extinction. The established extinction risk methods are global IUCN Red List status, vulnerability to fishing [23], and functional vulnerability. The color coded system highlights where each historically-exploited species was identified as threatened with local extinction by each established method. Normal font for agreement, italicized font for moderate agreement and bold font for disagreement.

Species	Global IUCN Red List status	Vulnerability to fishing	Functional vulnerability	
*Acanthopagrus berda*	**Least Concern**	*Moderate*		*Macro-invertivore*
*Alectis indica*	**Least Concern**	High		Piscivore / Macro-invertivore
*Atule mate*	**Not Assessed**	**Low**		*Macro-invertivore*
*Cephalopholis aurantia*	**Data Deficient**	*Moderate to High*		Piscivore / Macro-invertivore
*Dermatolepis striolata*	**Data Deficient**	High		Piscivore
*Epinephelus coeruleopunctatus*	**Least Concern**	High		Piscivore / Macro-invertivore
*Epinephelus lanceolatus*	Vulnerable	Very High		Piscivore
*Isurus paucus*	Vulnerable	Very High		Piscivore
*Lutjanus argentimaculatus*	**Least Concern**	High		Piscivore / Macro-invertivore
*Plectorhinchus plagiodesmus*	**Not Assessed**	High		Piscivore
*Plectorhinchus sordidus*	**Not Assessed**	*Low to Moderate*		Piscivore
*Plectropomus punctatus*	**Data Deficient**	High		Piscivore
*Plotosus limbatus*	**Not Assessed**	*Moderate*		Piscivore
*Rhabdosargus sarba*	**Not Assessed**	*Moderate*		Piscivore -/ Macro-invertivore
*Stegostoma fasciatum*	Endangered	Very High		Piscivore / Macro-invertivore

## Discussion

Given the research on species extinction and using historical data, we expected that carnivores would be the species threatened with local extinction and that our framework would corroborate the results of other well established extinction risk methods. As expected our results confirmed the first hypothesis but unexpectedly differed from the second hypothesis. In support of our species life histories hypothesis, we found all the species designated as possibly locally extinct by the two step framework are carnivores, specifically piscivores and macroinvertivores. However, the findings do not support the hypothesis that extinction risk methods identify similar species to the framework. The framework indicated divergent results for identifying reef species threatened with local extinction using disparate data versus those relying on established methods of extinction risk. The functional vulnerability method was a good predictor of species threatened with local extinction. Vulnerability to fishing traits was a moderate indicator of extinction risk while IUCN red list failed to identify many of the threatened species detected by our framework.

### Vulnerable species

At-risk species often elude detection because of the lack of species monitoring programs [59]. Surprisingly, our study identified a large number of well-known and conspicuous, rather than rare or inconspicuous, species as at-risk. Many of these species were identified as at risk as they were not sighted recently in fish transects and fish landings (Tables 1 and 2). A number of species identified by our method as threatened were commercially important carnivores. Additionally, piscivores and macro-invertivores are among the most vulnerable functional groups because they tend to be large bodied and exhibit long generation times [19, 24]. Finally, many carnivores are attracted to fishing bait, which can make even non-target or by-catch carnivores vulnerable to overexploitation [13]. The loss of carnivores found here reflects declines reported in other coral reefs and ecosystems [30, 33, 60, 61].

A number of the threatened species belong to the carnivorous family of Epinepheline groupers and Haemulidae sweetlips. Epinepheline groupers represent a valuable fishery resource that are inherently vulnerable to collapse due to fishing pressure [62,63]. One of their attributes that predispose them to intense fishing and population collapse is the formation of predictable spawning aggregations that fishers frequently learn to exploit. Sweetlips are less studied but are considered as vulnerable because both families share similar biological attributes such as large body size, extended longevity, late sexual maturation, and aggregating behavior, which correlate positively with extinction vulnerability [23, 26, 64]. A number of the other identified species are more difficult to classify in terms of the specific traits that make them vulnerable at the family level and may require more investigation to better understand the specific causes. Sweetlips and groupers are more easily observed and counted than some identified species and their higher abundance in fisheries closures indicates their vulnerability to even moderate levels of fishing [65, 66].

### Limitations of the method and data

Our study highlights some of the limitations in our methods and data for detecting the possible local extinction of species. The disparate data collection methods have different levels of reliability, sampling effort, and inherent biases [67]. Temporal resolution, for example, varied greatly among our data sources. Despite covering a 1,250-year time period, there were time and data gaps of 280 year (1500–1780) and 90 year (1900–1990). Spatial resolution varied among data sources. The archaeological records were confined to two sites and only represented captured species (Fig 1). In contrast to the archaeological records, the Kenyan contemporary catch assessments and underwater surveys were intensively and extensively sampled, but only for two decades.

Archaeological data were useful for exposing a number of threatened species, but cannot fully evaluate the extinction status of species arising during the contemporary period of intense fishing and climate change. The archaeological data had fewer species than the contemporary sources and there is now a new set of species that cannot be evaluated with historical data. For example, we did not find evidence that vulnerable functional groups such as bioeroding parrotfish (Scarine in the Labridae) were threatened. While some may argue that they have the ability to resist local extinction due to short generation times [66], we believe that many well-known species were not exploited during the historical period (750-1500CE) and cannot be evaluated using archeological data. Effective capture of parrotfish was made easier by the onset of modern fishing methods, including the use of scuba, snorkeling, and spear guns. If so, failure to detect vulnerable parrotfishes exposes a weakness of relying on archaeological data. In these cases, recent catch assessment records are needed to evaluate the status of parrotfish and similar species. Consequently, species subjected to recent exploitation will need their vulnerability evaluated by other methods, including life history considerations [23, 66], but also the need for continued monitoring of these species.

Factors affecting extinction risk, such as fishing intensity and climate are among influential factors not accounted for in this method. Past environments may have differed from current conditions and therefore influenced species composition. The archaeological data, for example, included periods during the Medieval Warm period when temperatures were increasing [31]. The modern assemblage was also likely to be influenced by warm and intensified climate, which alters species composition and vulnerabilities to extinction [5, 19,22,68,69]. Graham et al. [22], for example, noted that species differed in their vulnerabilities to climate disturbances and fishing intensity; small-bodied coral-dependent species were sensitive to climate disturbances while large-bodied and carnivorous were sensitive to fishing. Clearly, there is a need to further investigate the role that these changes and biases played in evaluating species vulnerabilities to extinction.

We are taking a precautionary approach in designating the 15 Kenyan reef species as threatened with local extinction rather than locally extinct. Incorrectly classifying a species as locally extinct can occur due to taxonomic uncertainty, observation error and process error [70]. Misclassification of extinction risk could lead to a false alarm, incorrectly classifying a species as being a conservation concern. Absence of a Kenyan species from the contemporary data sources does not necessarily reflect absence of a species from the region. Absence may represent a change in the behaviours of the fish or fishers’ records. Fishing effort, gear technology, efficiency, targeting, management, and market behaviours have changed and may have influenced presence/absence records [6,40,43,71–73]. However, the increasing fishing pressure and broad exploitation of reef fishes, irrespective of species and size, in modern Kenya makes these possibilities less likely [42].

Further evidence is required before classifying the Kenyan reef species as locally extinct. Further sources of uncertainty, natural variability and observation error, need to be accounted for. While contemporary catch assessment and underwater survey data were plentiful, observation error remains an uncertainty. Species absence needs to be verified by unexplored sources. For example, non-traditional data sources such as expedition reports, cookbook recipes and fisher knowledge have revealed the local and regional extinction of species [74,75]. Another source of uncertainty that needs to be accounted for is natural variability over time and space. Reconstructing the direction and rate of change in abundance provide a quantitative estimate of the decline and further evidence of local extinction [76–78].

Despite limitations, we demonstrated that combining data sources, using a two-step framework involving Bayesian extinction analysis, improved the detection of possibly locally extinct species. The archaeological data used greatly expanded the temporal depth and uncovered a number of threatened species not previously identified.

### Limitations of existing extinction risk assessments

Our study showed that limitations are evident in existing extinction risk methods. The IUCN Red List is the definitive index of extinction risk and has assessed a 7563 marine fishes [70]. However, the IUCN Red List assessments examine extinction risk at a global scale but apply the extinction risk independent of local factors. For example, three Kenyan species, which we identified as threatened with local extinction, were listed by the IUCN as “Least Concern”. The justification given was that despite local declines in certain areas of their range these species are common and abundant in other parts of their range [79]. This approach overlooks the possibility of local extinction.

Another limitation of the IUCN Red List assessment is the level of quantitative data required to carry out the assessment. This requirement is not always achievable, particularly for non-charismatic species or species with low commercial value. For these species records can be scarce. Our study identified three species as threatened with local extinction that the IUCN Red List categorised as “Data Deficient”. These species could go extinct before sufficient data is available for the IUCN Red List assessment.

Furthermore, the IUCN use a category “Not assessed” for species that haven’t been assessed for extinction risk. Six of the species we identified as threatened with local extinction are included in this IUCN category. There is a considerable risk that some of these six species will disappear before the IUCN can carry out an extinction risk assessment. Limitations to IUCN’s resources are understandable given the scale of their task; however relying solely on the IUCN Red List could result in these species going extinct without formal protection or recognition of their threatened status.

Similar to the IUCN Red List limitation concerning global level assessments, the vulnerability to fishing assessment method fails to reflect local differences in a species extinction risk, such as vulnerability to climate change [22]. Furthermore, recent work has shown that spatial variation in fishing needs to be taken into account when using life history traits [80]. These limitations of the vulnerability to fishing assessment method do not account for differential responses in species extinction risk at a local scale [81–83].

The other method, functional vulnerability, simplifies the species into groupings. While this is a useful indicator at an ecosystem level, it fails to produce a threatened list at the species scale [22]. This presents a challenge where incorporating functional vulnerability findings into conservation measures as these measures are typically set at a species level.

### Value and application of the framework

Our approach has advantages over existing extinction risk assessments as it can be used for data poor species and estimates probability of extinction at a species level. Our Kenyan case study highlights the importance of exploring and incorporating historical and disparate data sources. First the archaeological data produced a list of unaccounted for species, species that are no longer present in contemporary records. Secondly, extracting heterogeneous occurrence data from disparate sources enabled a reconstruction of species absence/presence over time overcoming the IUCN’s issue regarding minimum quantitative data requirements. The naturalists’ species list verified the occurrence of historically exploited species by taxonomic specialists between 1712 and 1940. Thirdly, the reconstruction of species occurrence showed the decline and possibly the local extinction of historically exploited species from Kenya’s reefs today.

The Bayesian extinction analysis proved a suitable approach for data poor species, i.e. when certain sightings are rare. Our framework succeeded in comparing heterogeneous data from disparate sources and accounted for uncertainty, in a demonstrable way. The Bayesian extinction analysis proved an effective model by explicitly accounting for several sources of uncertainty. Specifically the model accounted for the varied observation error among disparate data sources. The observation errors were as follows: true and false sightings and survey effort. The model accounted for the variability among types of sightings and survey effort. Another source of uncertainty that was accounted for was uncertainty around the prior, the prior belief that the species still occurs.

The framework used should be of value to conservation management efforts. The assessment of local extinction was carried out at a species level at a local scale which will allow easier application into species management policies and regulations. Furthermore, the framework proved an inexpensive and effective for identifying threatened species when historical data, particularly archaeological records, were available. Such assessments are especially critical for data- poor areas where inexpensive management advice is needed the most. Combining these data and applying Bayesian extinction risk methods can strengthen other species assessment efforts.

Local extinction rates are increasing among marine species [1,2] and here we have added fifteen new species potentially disappearing from Kenya’s reefs. Some of these species are likely to play important roles in ecological processes and services that may erode as they disappear. The first step towards avoiding local extinction is providing status information at a scale where decision-makers and managers can intervene. This is frequently at the local to national fisheries levels where policies can introduce size limits, protection of breeding seasons and locations, and outright bans to protect species most at risk.

## Supporting information

S1 Table List of keywords such as scientific and common names for species as well as changes in species names if their names changed. (PDF)

S2 Table List of fish families identified during underwater surveys. (PDF)

S3 Table List of historically exploited species and corresponding number for absence/presence tests. (PDF)

S1 Fig Quantifying the survey effort for each data source. (PDF)

S2 Fig The Excel spreadsheet shows an example of how the probability of local extinction was estimated using Bayesian extinction analysis using *Epinephelus coioides*. The spreadsheet accounts for different types of sightings, detectability and different types different surveys.
(PDF)

S1 Datasheet (XLSX)
