# Supplementary material for: Identifying species threatened with local extinction in tropical reef fisheries using historical reconstruction of species occurrence
Source: PLoS One. 2019 Feb 13;14(2):e0211224. doi: 10.1371/journal.pone.0211224 (PMC6373906; doi:10.1371/journal.pone.0211224)
Supplement: S1 Table — (PDF) [file pone.0211224.s001.pdf]

Supporting Information

S1 Table List of keywords such as scientific and common names for species as well as changes in species names if their names changed

Current scientific name	Common name	Original scientific name
Acanthopagrus berda	Seabream	*Chrysophrys berda*
Acanthurus lineatus	Lined surgeonfish	*Chaetodon lineatus*
Albula argentea		*Esox argenteus*
Alectis indica	Indian threadfish	*Alectis indicus*
Atule mate	Yellowtail scad	*Caranx mate*
Calotomus carolinus	Carolines parrotfish	*Callyodon carolinus*
Carangoides chrysophrys	Longnose trevally	*Caranx chrysophrys*
Carangoides fulvoguttatus	Yellowspotted trevally	*Scomber fulvoguttatus*
Caranx sexfasciatus	Bigeye trevally	
Cephalopholis aurantia	Golden hind	*Serranus aurantius*
Cetoscarus bicolor	Bicolour parrotfish	*Scarus bicolor*
Cheilio inermis	Cigar wrasse	*Labrus inermis*

Dermatolepis striolata	Smooth grouper	*Serranus striolatus*
Diagramma pictum	Painted sweetlips, grouper	*Perca picta*
Epinephelus coeruleopunctatus	White spotted grouper	*Holocentrus coeruleopunctatus*
Epinephelus coioides	Orange spotted grouper	*Bola coioides*
Epinephelus fasciatus	Golden grouper, Blacktip grouper	*Perca fasciata*
Epinephelus fuscoguttatus	Brown-marbled grouper	
Epinephelus lanceolatus	Giant grouper	*Holocentrus lanceolatus*
Epinephelus malabaricus	Malabar grouper	*Holocentrus malabaricus*
Gerres methueni		*Gerres longirostris*
Gymnothorax favagineus	Reticulated, laced moray	
Gymnothorax undulatus	Undulated moray	*Muraenophis undulata*
Hipposcarus harid	Longnose parrotfish	*Scarus harid*
Isurus paucus	Longfin mako, longfinned mako	*Isurus paucus*
Lactoria cornuta	Longhorn cowfish	*Ostracion cornutus*
Leptoscarus vaigiensis	Marbled parrotfish	*Scarus vaigiensis*
Lethrinus borbonicus	Snudnose emperor	

Lethrinus enigmaticus	Blackeye emperor	
Lethrinus harak	Blackspot emperor	*Sciaena harak*
Lethrinus lentjan	Redspot emperor	*Bodianus lentjan*
Lethrinus mahsena	Sky, mahsena emperor	*Sciaena mahsena*
Lethrinus microdon	Longnose, smalltooth emperor	
Lethrinus nebulosus	Spangled emperor	*Sciaena nebulosa*
Liza macrolepis	Large-scaled mullet	*Mugil macrolepis*
Lutjanus argentimaculatus	Mangrove red, river snapper	*Sciaena argentimaculata*
Lutjanus fulviflamma	Blackspot snapper	*Sciaena fulviflamma*
Lutjanus gibbus	Humpback snapper	*Sciaena gibba*
Naso hexacanthus	Sleek unicornfish	*Priodon hexacanthus*
Papilloculiceps longiceps	Tentacled flatfish	*Platycephalus longiceps*
Platax pinnatus	Dusky batfish	*Chaetodon pinnatus*
Plectorhinchus flavomaculatus	Lemonfish	*Diagramma flavomaculatum*
Plectorhinchus gaterinus	Blackspotted rubberlips	*Sciaena gaterina*
Plectorhinchus plagiodesmus	Barred rubberlip	

Plectorhinchus playfairi	Whitebarred rubberlips	
Plectorhinchus sordidus	Sordid rubberlips	*Diagramma sordidum*
Plectropomus punctatus	Marbled coralgrouper	*Plectropoma punctatum*
Plotosus limbatus	Dark fin eel catfish	
Pomacanthus chrysurus	Earspot angelfish	*Holacanthus chrysurus*
Pomadasys argenteus	Silver grunt	*Sciaena argentea*
Pomadasys maculatus	Saddle grunt	*Anthias maculatus*
Rhabdosargus sarba	Yellowfin, gold lined sea bream	*Sparus sarba*
Scarus ghobban	Blue-barred parrotfish	
Scarus niger	Dusky parrotfish	
Scarus psittacus	Palenose, common parrotfish	
Scarus rubroviolaceus	Bicolor, ember parrotfish	
Scarus russelii	Eclipse parrotfish	
Selar crumenophthalmus	Bigeye scad	*Scomber crumenophthalmus*
Siganus stellatus	Stellate rabbitfish, brown-spotted spinefoot	*Scarus stellatus*
Siganus sutor	Shoemaker spinefoot	*Amphacanthus sutor*

Sphyraena flavicauda	Yellowtail barracuda	
Sphyraena forsteri	Bigeye barracuda	
Stegostoma fasciatum	Zebra, leopard shark	*Squalus fasciatus*
Synchiropus stellatus	Starry dragonet	
Teixeirichthys jordani	Jordan's damsel	*Pomacentrus jordani*
Tylosurus acus acus	Atlantic needlefish	*Tylosurus acus*
Wetmorella albofasciata	Whitebanded sharpnose wrasse	
