# Supplementary material for: Identifying species threatened with local extinction in tropical reef fisheries using historical reconstruction of species occurrence
Source: PLoS One. 2019 Feb 13;14(2):e0211224. doi: 10.1371/journal.pone.0211224 (PMC6373906; doi:10.1371/journal.pone.0211224)
Supplement: S2 Table — (PDF) [file pone.0211224.s002.pdf]

S2 Table

List of fish families identified during underwater surveys

**Family**
Acanthuridae
Aulostomidae
Balistidae
Carangidae
Carcharhinidae
Chaetodontidae
Diodontidae
Fistularidae
Ginglymostomatidae
Haemulidae
Holocentridae
Labridae
Lethrinidae
Lutjanidae

Mullidae
Pempheridae
Penguipedidae
Pomacanthidae
Pomacentridae
Scaridae
Scorpaenidae
Siganidae
Sphyraenidae
Other
