# Supplementary material for: Identifying species threatened with local extinction in tropical reef fisheries using historical reconstruction of species occurrence
Source: PLoS One. 2019 Feb 13;14(2):e0211224. doi: 10.1371/journal.pone.0211224 (PMC6373906; doi:10.1371/journal.pone.0211224)
Supplement: S3 Table — (PDF) [file pone.0211224.s003.pdf]

**S5 Table List of historically exploited species and corresponding number for absence/presence tests.**

| Species number | Species | Absence/Present |
|---|---|---|
| 1 | *Epinephelus lanceolatus* | Absent |
| 2 | *Plotosus limbatus* | Absent |
| 3 | *Rhabdosargus sarba* | Absent |
| 4 | *Acanthopagrus berda* | Absent |
| 5 | *Epinephelus malabaricus* | Absent |
| 6 | *Gymnothorax favagineus* | Absent |
| 7 | *Pomadasys argenteus* | Absent |
| 8 | *Dermatolepis striolata* | Absent |
| 9 | *Plectorhinchus plagiodesmus* | Absent |
| 10 | *Tylosurus acus* | Absent |
| 11 | *Gymnothorax undulatus* | Absent |
| 12 | *Albula argentea* | Absent |
| 13 | *Atule mate* | Absent |
| 14 | *Isurus paucus* | Absent |
| 15 | *Lactoria cornuta* | Absent |
| 16 | *Lethrinus enigmaticus* | Present |
| 17 | *Liza macrolepis* | Absent |
| 18 | *Platax pinnatus* | Absent |
| 19 | *Leptoscarus vaigiensis* | Absent |
| 20 | *Plectorhinchus* | Absent |

| | | |
|---|---|---|
| | *flavomaculatus* | |
| 21 | *Scarus ghobban* | Present |
| 22 | *Siganus sutor* | Absent |
| 23 | *Pomadasys maculatus* | Absent |
| 24 | *Cheilio inermis* | Present |
| 25 | *Acanthurus lineatus* | Present |
| 26 | *Alectis indica* | Absent |
| 27 | *Lutjanus fulviflamma* | Present |
| 28 | *Epinephelus caeruleopunctatus* | Absent |
| 29 | *Papilloculiceps longiceps* | Present |
| 30 | *Plectorhinchus gaterinus* | Present |
| 31 | *Scarus niger* | Present |
| 32 | *Gerres longirostris* | Present |
| 33 | *Scarus rubroviolaceus* | Present |
| 34 | *Cephalopholis aurantia* | Present |
| 35 | *Plectropomus punctatus* | Absent |
| 36 | *Plectorhinchus sordidus* | Absent |
| 37 | *Siganus stellatus* | Present |
| 38 | *Lethrinus nebulosus* | Present |
| 39 | *Lutjanus argentimaculatus* | Absent |
| 40 | *Lethrinus mahsena* | Present |
| 41 | *Epinephelus coioides* | Absent |
| 42 | *Naso hexacanthus* | Absent |

| | | |
|---|---|---|
| 43 | *Sphyraena barracuda* | Present |
| 44 | *Calotomus carolinus* | Present |
| 45 | *Caranx sexfasciatus* | Present |
| 46 | *Stegastoma fasciatum* | Absent |
| 47 | *Variola louti* | Present |
| 48 | *Lethrinus microdon* | Present |
| 49 | *Plectorhinchus playfairi* | Present |
| 50 | *Pomacanthus chrysurus* | Present |
| 51 | *Epinephelus fuscoguttatus* | Present |
| 52 | *Selar crumenophthalmus* | Absent |
| 53 | *Lethrinus harak* | Present |
| 54 | *Scarus psittacus* | Present |
| 55 | *Sphyraena flavicauda* | Present |
| 56 | *Carangoides fulvoguttatus* | Present |
| 57 | *Hipposcarus harid* | Present |
| 58 | *Lethrinus lentjan* | Present |
| 59 | *Scarus russelii* | Absent |
| 60 | *Carangoides chrysophrys* | Present |
| 61 | *Cetoscarus stronglyocentrotus* | Present |
| 62 | *Diagramma pictum* | Absent |
| 63 | *Epinephelus fasciatus* | Present |
| 64 | *Lethrinus borbonicus* | Present |
| 65 | *Lutjanus gibbus* | Present |
| 66 | *Synchiropus stellatus* | Present |

| | | |
|---|---|---|
| 67 | *Teixeirichthys jordani* | Present |
