# Supplementary material for: Identifying species threatened with local extinction in tropical reef fisheries using historical reconstruction of species occurrence
Source: PLoS One. 2019 Feb 13;14(2):e0211224. doi: 10.1371/journal.pone.0211224 (PMC6373906; doi:10.1371/journal.pone.0211224)
Supplement: S1 Fig — (PDF) [file pone.0211224.s004.pdf]

S1 Fig

Quantifying the survey effort for each data source

The likelihood of sighting a species based on the contemporary catch assessments was 0.70-0.90 detectability if extant and 0.00-0.70 if extinct. Catch assessment detectability was based on surveys being conducted annually throughout seasons, the sampling encompassed the entire coastline of Kenya, indiscriminate sampling of species landed and highly skilled observers.

The likelihood of sighting a species based on the naturalist's species lists was 0.40-0.60 detectability if extant and 0.00-0.60 if extinct. For Naturalist's species lists the detectability was based on extensive surveys conducted by naturalists, expert naturalists surveyed the entire Kenyan coastline and habitats, the distinctiveness of the fish species and highly skilled observer.

The likelihood of sighting a species based on the underwater surveys was 0.60-0.80 detectability if extant and 0.00-0.60 if extinct. For underwater surveys the detectability was based on the monitoring being conducted annually, one expert observer conducted the surveys, the surveys only covered inshore coral reefs, the distinctiveness of the fish species and highly skilled observer.

The likelihood of sighting a species was not quantified for the archaeological records or naturalists' species lists because no specific surveys were conducted.
