# Supplementary material for: Identifying species threatened with local extinction in tropical reef fisheries using historical reconstruction of species occurrence
Source: PLoS One. 2019 Feb 13;14(2):e0211224. doi: 10.1371/journal.pone.0211224 (PMC6373906; doi:10.1371/journal.pone.0211224)
Supplement: S2 Fig — The spreadsheet accounts for different types of sightings, detectability and different types different surveys. (PDF) [file pone.0211224.s005.pdf]

S4 Fig

The Excel spreadsheet for estimating the probability of local extinction with Bayesian extinction analysis using Epinephelus coioides as an example. The spreadsheet accounts for different types of sightings, detectability and different types different surveys.

Sighting Record

Years	Date	Certain	Survey 1	Survey 2	Survey 3	A	B	C
1	750	0				0	0	0
2	751	0				0	0	0
3	752	0				0	0	0
4	753	0				0	0	0
5	754	0				0	0	0
6	755	0				0	0	0
7	756	0				0	0	0
8	757	0				0	0	0
9	758	0				0	0	0
10	759	0				0	0	0
11	760	0				0	0	0
12	761	0				0	0	0
13	762	0				0	0	0
14	763	0				0	0	0
15	764	0				0	0	0
16	765	0				0	0	0
17	766	0				0	0	0
18	767	0				0	0	0
19	768	0				0	0	0
20	769	0				0	0	0
21	770	0				0	0	0
22	771	0				0	0	0
23	772	0				0	0	0
24	773	0				0	0	0
25	774	0				0	0	0
26	775	0				0	0	0
27	776	0				0	0	0
28	777	0				0	0	0
29	778	0				0	0	0
30	779	0				0	0	0
31	780	0				0	0	0
32	781	0				0	0	0
33	782	0				0	0	0
34	783	0				0	0	0
35	784	0				0	0	0
36	785	0				0	0	0
37	786	0				0	0	0
38	787	0				0	0	0

Detectability bounds

Assume probability of seeing a		
SPECIMEN	0.0000	0.0200
UNCERTAIN A	0.0105	0.0111
UNCERTAIN B	0.0111	0.0125
UNCERTAIN C	0.0111	0.0125
UNCERTAIN D		
UNCERTAIN E		

given the species is **EXTANT**, is uniformly distributed between the bounds in green.

False detectability bounds

Assume probability of seeing a		
SPECIMEN	0	0
UNCERTAIN A	0.0005	0.0011
UNCERTAIN B	0.0011	0.0025
UNCERTAIN C	0.0011	0.0025
UNCERTAIN D		
UNCERTAIN E		

given the species is **EXTINCT**, is uniformly distributed between the bounds in green.

Prior bounds

Prior for extant (P(XT))	0.177777778	0.377778
Prior for extinct in a year (PE)	0.0016	0.0012

Survey Quality bounds

Probability of seeing the species given that it is **EXTANT** is between

Survey 1	0.7	0.9
Survey 2	0.4	0.6
Survey 3	0.6	0.8

Probability of seeing the species given that it is **EXTINCT** is between

Survey 1	0	0.7
Survey 2	0	0.4
Survey 3	0	0.6

Number of years (T)	720
Years until last sighting (TN)	200
Number of sightings (S)	2
**Probability extant**	**0.3112**
(Bayes factor method)	0.4718

Only write in Green cells please.

39	788	0		0	0	0
40	789	0		0	0	0
41	790	0		0	0	0
42	791	0		0	0	0
43	792	0		0	0	0
44	793	0		0	0	0
45	794	0		0	0	0
46	795	0		0	0	0
47	796	0		0	0	0
48	797	0		0	0	0
49	798	0		0	0	0
50	799	0		0	0	0
51	800	0		0	0	0
52	801	0		0	0	0
53	802	0		0	0	0
54	803	0		0	0	0
55	804	0		0	0	0
56	805	0		0	0	0
57	806	0		0	0	0
58	807	0		0	0	0
59	808	0		0	0	0
60	809	0		0	0	0
61	810	0		0	0	0
62	811	0		0	0	0
63	812	0		0	0	0
64	813	0		0	0	0
65	814	0		0	0	0
66	815	0		0	0	0
67	816	0		0	0	0
68	817	0		0	0	0
69	818	0		0	0	0
70	819	0		0	0	0
71	820	0		0	0	0
72	821	0		0	0	0
73	822	0		0	0	0
74	823	0		0	0	0
75	824	0		0	0	0
76	825	0		0	0	0
77	826	0		0	0	0
78	827	0		0	0	0
79	828	0		0	0	0
80	829	0		0	0	0
81	830	0		0	0	0

82	831	0		0	0	0
83	832	0		0	0	0
84	833	0		0	0	0
85	834	0		0	0	0
86	835	0		0	0	0
87	836	0		0	0	0
88	837	0		0	0	0
89	838	0		0	0	0
90	839	0		0	0	0
91	840	0		0	0	0
92	841	0		0	0	0
93	842	0		0	0	0
94	843	0		0	0	0
95	844	0		0	0	0
96	845	0		0	0	0
97	846	0		0	0	0
98	847	0		0	0	0
99	848	0		0	0	0
100	849	0		0	0	0
101	850	0		0	0	0
102	851	0		0	0	0
103	852	0		0	0	0
104	853	0		0	0	0
105	854	0		0	0	0
106	855	0		0	0	0
107	856	0		0	0	0
108	857	0		0	0	0
109	858	0		0	0	0
110	859	0		0	0	0
111	860	0		0	0	0
112	861	0		0	0	0
113	862	0		0	0	0
114	863	0		0	0	0
115	864	0		0	0	0
116	865	0		0	0	0
117	866	0		0	0	0
118	867	0		0	0	0
119	868	0		0	0	0
120	869	0		0	0	0
121	870	0		0	0	0
122	871	0		0	0	0
123	872	0		0	0	0
124	873	0		0	0	0

125	874	0		0	0	0
126	875	0		0	0	0
127	876	0		0	0	0
128	877	0		0	0	0
129	878	0		0	0	0
130	879	0		0	0	0
131	880	0		0	0	0
132	881	0		0	0	0
133	882	0		0	0	0
134	883	0		0	0	0
135	884	0		0	0	0
136	885	0		0	0	0
137	886	0		0	0	0
138	887	0		0	0	0
139	888	0		0	0	0
140	889	0		0	0	0
141	890	0		0	0	0
142	891	0		0	0	0
143	892	0		0	0	0
144	893	0		0	0	0
145	894	0		0	0	0
146	895	0		0	0	0
147	896	0		0	0	0
148	897	0		0	0	0
149	898	0		0	0	0
150	899	0		0	0	0
151	900	0		0	0	0
152	901	0		0	0	0
153	902	0		0	0	0
154	903	0		0	0	0
155	904	0		0	0	0
156	905	0		0	0	0
157	906	0		0	0	0
158	907	0		0	0	0
159	908	0		0	0	0
160	909	0		0	0	0
161	910	0		0	0	0
162	911	0		0	0	0
163	912	0		0	0	0
164	913	0		0	0	0
165	914	0		0	0	0
166	915	0		0	0	0
167	916	0		0	0	0

168	917	0		0	0	0
169	918	0		0	0	0
170	919	0		0	0	0
171	920	0		0	0	0
172	921	0		0	0	0
173	922	0		0	0	0
174	923	0		0	0	0
175	924	0		0	0	0
176	925	0		0	0	0
177	926	0		0	0	0
178	927	0		0	0	0
179	928	0		0	0	0
180	929	0		0	0	0
181	930	0		0	0	0
182	931	0		0	0	0
183	932	0		0	0	0
184	933	0		0	0	0
185	934	0		0	0	0
186	935	0		0	0	0
187	936	0		0	0	0
188	937	0		0	0	0
189	938	0		0	0	0
190	939	0		0	0	0
191	940	0		0	0	0
192	941	0		0	0	0
193	942	0		0	0	0
194	943	0		0	0	0
195	944	0		0	0	0
196	945	0		0	0	0
197	946	0		0	0	0
198	947	0		0	0	0
199	948	0		0	0	0
200	949	0		0	0	0
201	950	0		0	0	0
202	951	0		0	0	0
203	952	0		0	0	0
204	953	0		0	0	0
205	954	0		0	0	0
206	955	0		0	0	0
207	956	0		0	0	0
208	957	0		0	0	0
209	958	0		0	0	0
210	959	0		0	0	0

211	960	0		0	0	0
212	961	0		0	0	0
213	962	0		0	0	0
214	963	0		0	0	0
215	964	0		0	0	0
216	965	0		0	0	0
217	966	0		0	0	0
218	967	0		0	0	0
219	968	0		0	0	0
220	969	0		0	0	0
221	970	0		0	0	0
222	971	0		0	0	0
223	972	0		0	0	0
224	973	0		0	0	0
225	974	0		0	0	0
226	975	0		0	0	0
227	976	0		0	0	0
228	977	0		0	0	0
229	978	0		0	0	0
230	979	0		0	0	0
231	980	0		0	0	0
232	981	0		0	0	0
233	982	0		0	0	0
234	983	0		0	0	0
235	984	0		0	0	0
236	985	0		0	0	0
237	986	0		0	0	0
238	987	0		0	0	0
239	988	0		0	0	0
240	989	0		0	0	0
241	990	0		0	0	0
242	991	0		0	0	0
243	992	0		0	0	0
244	993	0		0	0	0
245	994	0		0	0	0
246	995	0		0	0	0
247	996	0		0	0	0
248	997	0		0	0	0
249	998	0		0	0	0
250	999	0		0	0	0
251	1000	0		0	0	0
252	1001	0		0	0	0
253	1002	0		0	0	0

254	1003	0		0	0	0
255	1004	0		0	0	0
256	1005	0		0	0	0
257	1006	0		0	0	0
258	1007	0		0	0	0
259	1008	0		0	0	0
260	1009	0		0	0	0
261	1010	0		0	0	0
262	1011	0		0	0	0
263	1012	0		0	0	0
264	1013	0		0	0	0
265	1014	0		0	0	0
266	1015	0		0	0	0
267	1016	0		0	0	0
268	1017	0		0	0	0
269	1018	0		0	0	0
270	1019	0		0	0	0
271	1020	0		0	0	0
272	1021	0		0	0	0
273	1022	0		0	0	0
274	1023	0		0	0	0
275	1024	0		0	0	0
276	1025	0		0	0	0
277	1026	0		0	0	0
278	1027	0		0	0	0
279	1028	0		0	0	0
280	1029	0		0	0	0
281	1030	0		0	0	0
282	1031	0		0	0	0
283	1032	0		0	0	0
284	1033	0		0	0	0
285	1034	0		0	0	0
286	1035	0		0	0	0
287	1036	0		0	0	0
288	1037	0		0	0	0
289	1038	0		0	0	0
290	1039	0		0	0	0
291	1040	0		0	0	0
292	1041	0		0	0	0
293	1042	0		0	0	0
294	1043	0		0	0	0
295	1044	0		0	0	0
296	1045	0		0	0	0

297	1046	0		0	0	0
298	1047	0		0	0	0
299	1048	0		0	0	0
300	1049	0		0	0	0
301	1050	0		0	0	0
302	1051	0		0	0	0
303	1052	0		0	0	0
304	1053	0		0	0	0
305	1054	0		0	0	0
306	1055	0		0	0	0
307	1056	0		0	0	0
308	1057	0		0	0	0
309	1058	0		0	0	0
310	1059	0		0	0	0
311	1060	0		0	0	0
312	1061	0		0	0	0
313	1062	0		0	0	0
314	1063	0		0	0	0
315	1064	0		0	0	0
316	1065	0		0	0	0
317	1066	0		0	0	0
318	1067	0		0	0	0
319	1068	0		0	0	0
320	1069	0		0	0	0
321	1070	0		0	0	0
322	1071	0		0	0	0
323	1072	0		0	0	0
324	1073	0		0	0	0
325	1074	0		0	0	0
326	1075	0		0	0	0
327	1076	0		0	0	0
328	1077	0		0	0	0
329	1078	0		0	0	0
330	1079	0		0	0	0
331	1080	0		0	0	0
332	1081	0		0	0	0
333	1082	0		0	0	0
334	1083	0		0	0	0
335	1084	0		0	0	0
336	1085	0		0	0	0
337	1086	0		0	0	0
338	1087	0		0	0	0
339	1088	0		0	0	0

340	1089	0		0	0	0
341	1090	0		0	0	0
342	1091	0		0	0	0
343	1092	0		0	0	0
344	1093	0		0	0	0
345	1094	0		0	0	0
346	1095	0		0	0	0
347	1096	0		0	0	0
348	1097	0		0	0	0
349	1098	0		0	0	0
350	1099	0		0	0	0
351	1100	0		0	0	0
352	1101	0		0	0	0
353	1102	0		0	0	0
354	1103	0		0	0	0
355	1104	0		0	0	0
356	1105	0		0	0	0
357	1106	0		0	0	0
358	1107	0		0	0	0
359	1108	0		0	0	0
360	1109	0		0	0	0
361	1110	0		0	0	0
362	1111	0		0	0	0
363	1112	0		0	0	0
364	1113	0		0	0	0
365	1114	0		0	0	0
366	1115	0		0	0	0
367	1116	0		0	0	0
368	1117	0		0	0	0
369	1118	0		0	0	0
370	1119	0		0	0	0
371	1120	0		0	0	0
372	1121	0		0	0	0
373	1122	0		0	0	0
374	1123	0		0	0	0
375	1124	0		0	0	0
376	1125	0		0	0	0
377	1126	0		0	0	0
378	1127	0		0	0	0
379	1128	0		0	0	0
380	1129	0		0	0	0
381	1130	0		0	0	0
382	1131	0		0	0	0

383	1132	0		0	0	0
384	1133	0		0	0	0
385	1134	0		0	0	0
386	1135	0		0	0	0
387	1136	0		0	0	0
388	1137	0		0	0	0
389	1138	0		0	0	0
390	1139	0		0	0	0
391	1140	0		0	0	0
392	1141	0		0	0	0
393	1142	0		0	0	0
394	1143	0		0	0	0
395	1144	0		0	0	0
396	1145	0		0	0	0
397	1146	0		0	0	0
398	1147	0		0	0	0
399	1148	0		0	0	0
400	1149	0		0	0	0
401	1150	0		0	0	0
402	1151	0		0	0	0
403	1152	0		0	0	0
404	1153	0		0	0	0
405	1154	0		0	0	0
406	1155	0		0	0	0
407	1156	0		0	0	0
408	1157	0		0	0	0
409	1158	0		0	0	0
410	1159	0		0	0	0
411	1160	0		0	0	0
412	1161	0		0	0	0
413	1162	0		0	0	0
414	1163	0		0	0	0
415	1164	0		0	0	0
416	1165	0		0	0	0
417	1166	0		0	0	0
418	1167	0		0	0	0
419	1168	0		0	0	0
420	1169	0		0	0	0
421	1170	0		0	0	0
422	1171	0		0	0	0
423	1172	0		0	0	0
424	1173	0		0	0	0
425	1174	0		0	0	0

426	1175	0		0	0	0
427	1176	0		0	0	0
428	1177	0		0	0	0
429	1178	0		0	0	0
430	1179	0		0	0	0
431	1180	0		0	0	0
432	1181	0		0	0	0
433	1182	0		0	0	0
434	1183	0		0	0	0
435	1184	0		0	0	0
436	1185	0		0	0	0
437	1186	0		0	0	0
438	1187	0		0	0	0
439	1188	0		0	0	0
440	1189	0		0	0	0
441	1190	0		0	0	0
442	1191	0		0	0	0
443	1192	0		0	0	0
444	1193	0		0	0	0
445	1194	0		0	0	0
446	1195	0		0	0	0
447	1196	0		0	0	0
448	1197	0		0	0	0
449	1198	0		0	0	0
450	1199	0		0	0	0
451	1200	0		0	0	0
452	1201	0		0	0	0
453	1202	0		0	0	0
454	1203	0		0	0	0
455	1204	0		0	0	0
456	1205	0		0	0	0
457	1206	0		0	0	0
458	1207	0		0	0	0
459	1208	0		0	0	0
460	1209	0		0	0	0
461	1210	0		0	0	0
462	1211	0		0	0	0
463	1212	0		0	0	0
464	1213	0		0	0	0
465	1214	0		0	0	0
466	1215	0		0	0	0
467	1216	0		0	0	0
468	1217	0		0	0	0

469	1218	0		0	0	0
470	1219	0		0	0	0
471	1220	0		0	0	0
472	1221	0		0	0	0
473	1222	0		0	0	0
474	1223	0		0	0	0
475	1224	0		0	0	0
476	1225	0		0	0	0
477	1226	0		0	0	0
478	1227	0		0	0	0
479	1228	0		0	0	0
480	1229	0		0	0	0
481	1230	0		0	0	0
482	1231	0		0	0	0
483	1232	0		0	0	0
484	1233	0		0	0	0
485	1234	0		0	0	0
486	1235	0		0	0	0
487	1236	0		0	0	0
488	1237	0		0	0	0
489	1238	0		0	0	0
490	1239	0		0	0	0
491	1240	0		0	0	0
492	1241	0		0	0	0
493	1242	0		0	0	0
494	1243	0		0	0	0
495	1244	0		0	0	0
496	1245	0		0	0	0
497	1246	0		0	0	0
498	1247	0		0	0	0
499	1248	0		0	0	0
500	1249	0		0	0	0
501	1250	0		0	0	0
502	1251	0		0	0	0
503	1252	0		0	0	0
504	1253	0		0	0	0
505	1254	0		0	0	0
506	1255	0		0	0	0
507	1256	0		0	0	0
508	1257	0		0	0	0
509	1258	0		0	0	0
510	1259	0		0	0	0
511	1260	0		0	0	0

512	1261	0		0	0	0
513	1262	0		0	0	0
514	1263	0		0	0	0
515	1264	0		0	0	0
516	1265	0		0	0	0
517	1266	0		0	0	0
518	1267	0		0	0	0
519	1268	0		0	0	0
520	1269	0		0	0	0
521	1270	0		0	0	0
522	1271	0		0	0	0
523	1272	0		0	0	0
524	1273	0		0	0	0
525	1274	0		0	0	0
526	1275	0		0	0	0
527	1276	0		0	0	0
528	1277	0		0	0	0
529	1278	0		0	0	0
530	1279	0		0	0	0
531	1280	0		0	0	0
532	1281	0		0	0	0
533	1282	0		0	0	0
534	1283	0		0	0	0
535	1284	0		0	0	0
536	1285	0		0	0	0
537	1286	0		0	0	0
538	1287	0		0	0	0
539	1288	0		0	0	0
540	1289	0		0	0	0
541	1290	0		0	0	0
542	1291	0		0	0	0
543	1292	0		0	0	0
544	1293	0		0	0	0
545	1294	0		0	0	0
546	1295	0		0	0	0
547	1296	0		0	0	0
548	1297	0		0	0	0
549	1298	0		0	0	0
550	1299	0		0	0	0
551	1300	1		0	0	0
552	1301	0		0	0	0
553	1302	0		0	0	0
554	1303	0		0	0	0

555	1304	0		0	0	0
556	1305	0		0	0	0
557	1306	0		0	0	0
558	1307	0		0	0	0
559	1308	0		0	0	0
560	1309	0		0	0	0
561	1310	0		0	0	0
562	1311	0		0	0	0
563	1312	0		0	0	0
564	1313	0		0	0	0
565	1314	0		0	0	0
566	1315	0		0	0	0
567	1316	0		0	0	0
568	1317	0		0	0	0
569	1318	0		0	0	0
570	1319	0		0	0	0
571	1320	0		0	0	0
572	1321	0		0	0	0
573	1322	0		0	0	0
574	1323	0		0	0	0
575	1324	0		0	0	0
576	1325	0		0	0	0
577	1326	0		0	0	0
578	1327	0		0	0	0
579	1328	0		0	0	0
580	1329	0		0	0	0
581	1330	0		0	0	0
582	1331	0		0	0	0
583	1332	0		0	0	0
584	1333	0		0	0	0
585	1334	0		0	0	0
586	1335	0		0	0	0
587	1336	0		0	0	0
588	1337	0		0	0	0
589	1338	0		0	0	0
590	1339	0		0	0	0
591	1340	0		0	0	0
592	1341	0		0	0	0
593	1342	0		0	0	0
594	1343	0		0	0	0
595	1344	0		0	0	0
596	1345	0		0	0	0
597	1346	0		0	0	0

598	1347	0		0	0	0
599	1348	0		0	0	0
600	1349	0		0	0	0
601	1350	0		0	0	0
602	1351	0		0	0	0
603	1352	0		0	0	0
604	1353	0		0	0	0
605	1354	0		0	0	0
606	1355	0		0	0	0
607	1356	0		0	0	0
608	1357	0		0	0	0
609	1358	0		0	0	0
610	1359	0		0	0	0
611	1360	0		0	0	0
612	1361	0		0	0	0
613	1362	0		0	0	0
614	1363	0		0	0	0
615	1364	0		0	0	0
616	1365	0		0	0	0
617	1366	0		0	0	0
618	1367	0		0	0	0
619	1368	0		0	0	0
620	1369	0		0	0	0
621	1370	0		0	0	0
622	1371	0		0	0	0
623	1372	0		0	0	0
624	1373	0		0	0	0
625	1374	0		0	0	0
626	1375	0		0	0	0
627	1376	0		0	0	0
628	1377	0		0	0	0
629	1378	0		0	0	0
630	1379	0		0	0	0
631	1380	0		0	0	0
632	1381	0		0	0	0
633	1382	0		0	0	0
634	1383	0		0	0	0
635	1384	0		0	0	0
636	1385	0		0	0	0
637	1386	0		0	0	0
638	1387	0		0	0	0
639	1388	0		0	0	0
640	1389	0		0	0	0

641	1390	0		0	0	0
642	1391	0		0	0	0
643	1392	0		0	0	0
644	1393	0		0	0	0
645	1394	0		0	0	0
646	1395	0		0	0	0
647	1396	0		0	0	0
648	1397	0		0	0	0
649	1398	0		0	0	0
650	1399	0		0	0	0
651	1400	0		0	0	0
652	1401	0		0	0	0
653	1402	0		0	0	0
654	1403	0		0	0	0
655	1404	0		0	0	0
656	1405	0		0	0	0
657	1406	0		0	0	0
658	1407	0		0	0	0
659	1408	0		0	0	0
660	1409	0		0	0	0
661	1410	0		0	0	0
662	1411	0		0	0	0
663	1412	0		0	0	0
664	1413	0		0	0	0
665	1414	0		0	0	0
666	1415	0		0	0	0
667	1416	0		0	0	0
668	1417	0		0	0	0
669	1418	0		0	0	0
670	1419	0		0	0	0
671	1420	0		0	0	0
672	1421	0		0	0	0
673	1422	0		0	0	0
674	1423	0		0	0	0
675	1424	0		0	0	0
676	1425	0		0	0	0
677	1426	0		0	0	0
678	1427	0		0	0	0
679	1428	0		0	0	0
680	1429	0		0	0	0
681	1430	0		0	0	0
682	1431	0		0	0	0
683	1432	0		0	0	0

684	1433	0		0	0	0
685	1434	0		0	0	0
686	1435	0		0	0	0
687	1436	0		0	0	0
688	1437	0		0	0	0
689	1438	0		0	0	0
690	1439	0		0	0	0
691	1440	0		0	0	0
692	1441	0		0	0	0
693	1442	0		0	0	0
694	1443	0		0	0	0
695	1444	0		0	0	0
696	1445	0		0	0	0
697	1446	0		0	0	0
698	1447	0		0	0	0
699	1448	0		0	0	0
700	1449	0		0	0	0
701	1450	0		0	0	0
702	1451	0		0	0	0
703	1452	0		0	0	0
704	1453	0		0	0	0
705	1454	0		0	0	0
706	1455	0		0	0	0
707	1456	0		0	0	0
708	1457	0		0	0	0
709	1458	0		0	0	0
710	1459	0		0	0	0
711	1460	0		0	0	0
712	1461	0		0	0	0
713	1462	0		0	0	0
714	1463	0		0	0	0
715	1464	0		0	0	0
716	1465	0		0	0	0
717	1466	0		0	0	0
718	1467	0		0	0	0
719	1468	0		0	0	0
720	1469	0		0	0	0
721	1470	0		0	0	0
722	1471	0		0	0	0
723	1472	0		0	0	0
724	1473	0		0	0	0
725	1474	0		0	0	0
726	1475	0		0	0	0

727	1476	0		0	0	0
728	1477	0		0	0	0
729	1478	0		0	0	0
730	1479	0		0	0	0
731	1480	0		0	0	0
732	1481	0		0	0	0
733	1482	0		0	0	0
734	1483	0		0	0	0
735	1484	0		0	0	0
736	1485	0		0	0	0
737	1486	0		0	0	0
738	1487	0		0	0	0
739	1488	0		0	0	0
740	1489	0		0	0	0
741	1490	0		0	0	0
742	1491	0		0	0	0
743	1492	0		0	0	0
744	1493	0		0	0	0
745	1494	0		0	0	0
746	1495	0		0	0	0
747	1496	0		0	0	0
748	1497	0		0	0	0
749	1498	0		0	0	0
750	1499	0		0	0	0
751	1500	1		0	0	0
752	1501	0		0	0	0
753	1502	0		0	0	0
754	1503	0		0	0	0
755	1504	0		0	0	0
756	1505	0		0	0	0
757	1506	0		0	0	0
758	1507	0		0	0	0
759	1508	0		0	0	0
760	1509	0		0	0	0
761	1510	0		0	0	0
762	1511	0		0	0	0
763	1512	0		0	0	0
764	1513	0		0	0	0
765	1514	0		0	0	0
766	1515	0		0	0	0
767	1516	0		0	0	0
768	1517	0		0	0	0
769	1518	0		0	0	0

770	1519	0		0	0	0
771	1520	0		0	0	0
772	1521	0		0	0	0
773	1522	0		0	0	0
774	1523	0		0	0	0
775	1524	0		0	0	0
776	1525	0		0	0	0
777	1526	0		0	0	0
778	1527	0		0	0	0
779	1528	0		0	0	0
780	1529	0		0	0	0
781	1530	0		0	0	0
782	1531	0		0	0	0
783	1532	0		0	0	0
784	1533	0		0	0	0
785	1534	0		0	0	0
786	1535	0		0	0	0
787	1536	0		0	0	0
788	1537	0		0	0	0
789	1538	0		0	0	0
790	1539	0		0	0	0
791	1540	0		0	0	0
792	1541	0		0	0	0
793	1542	0		0	0	0
794	1543	0		0	0	0
795	1544	0		0	0	0
796	1545	0		0	0	0
797	1546	0		0	0	0
798	1547	0		0	0	0
799	1548	0		0	0	0
800	1549	0		0	0	0
801	1550	0		0	0	0
802	1551	0		0	0	0
803	1552	0		0	0	0
804	1553	0		0	0	0
805	1554	0		0	0	0
806	1555	0		0	0	0
807	1556	0		0	0	0
808	1557	0		0	0	0
809	1558	0		0	0	0
810	1559	0		0	0	0
811	1560	0		0	0	0
812	1561	0		0	0	0

813	1562	0		0	0	0
814	1563	0		0	0	0
815	1564	0		0	0	0
816	1565	0		0	0	0
817	1566	0		0	0	0
818	1567	0		0	0	0
819	1568	0		0	0	0
820	1569	0		0	0	0
821	1570	0		0	0	0
822	1571	0		0	0	0
823	1572	0		0	0	0
824	1573	0		0	0	0
825	1574	0		0	0	0
826	1575	0		0	0	0
827	1576	0		0	0	0
828	1577	0		0	0	0
829	1578	0		0	0	0
830	1579	0		0	0	0
831	1580	0		0	0	0
832	1581	0		0	0	0
833	1582	0		0	0	0
834	1583	0		0	0	0
835	1584	0		0	0	0
836	1585	0		0	0	0
837	1586	0		0	0	0
838	1587	0		0	0	0
839	1588	0		0	0	0
840	1589	0		0	0	0
841	1590	0		0	0	0
842	1591	0		0	0	0
843	1592	0		0	0	0
844	1593	0		0	0	0
845	1594	0		0	0	0
846	1595	0		0	0	0
847	1596	0		0	0	0
848	1597	0		0	0	0
849	1598	0		0	0	0
850	1599	0		0	0	0
851	1600	0		0	0	0
852	1601	0		0	0	0
853	1602	0		0	0	0
854	1603	0		0	0	0
855	1604	0		0	0	0

856	1605	0		0	0	0
857	1606	0		0	0	0
858	1607	0		0	0	0
859	1608	0		0	0	0
860	1609	0		0	0	0
861	1610	0		0	0	0
862	1611	0		0	0	0
863	1612	0		0	0	0
864	1613	0		0	0	0
865	1614	0		0	0	0
866	1615	0		0	0	0
867	1616	0		0	0	0
868	1617	0		0	0	0
869	1618	0		0	0	0
870	1619	0		0	0	0
871	1620	0		0	0	0
872	1621	0		0	0	0
873	1622	0		0	0	0
874	1623	0		0	0	0
875	1624	0		0	0	0
876	1625	0		0	0	0
877	1626	0		0	0	0
878	1627	0		0	0	0
879	1628	0		0	0	0
880	1629	0		0	0	0
881	1630	0		0	0	0
882	1631	0		0	0	0
883	1632	0		0	0	0
884	1633	0		0	0	0
885	1634	0		0	0	0
886	1635	0		0	0	0
887	1636	0		0	0	0
888	1637	0		0	0	0
889	1638	0		0	0	0
890	1639	0		0	0	0
891	1640	0		0	0	0
892	1641	0		0	0	0
893	1642	0		0	0	0
894	1643	0		0	0	0
895	1644	0		0	0	0
896	1645	0		0	0	0
897	1646	0		0	0	0
898	1647	0		0	0	0

899	1648	0		0	0	0
900	1649	0		0	0	0
901	1650	0		0	0	0
902	1651	0		0	0	0
903	1652	0		0	0	0
904	1653	0		0	0	0
905	1654	0		0	0	0
906	1655	0		0	0	0
907	1656	0		0	0	0
908	1657	0		0	0	0
909	1658	0		0	0	0
910	1659	0		0	0	0
911	1660	0		0	0	0
912	1661	0		0	0	0
913	1662	0		0	0	0
914	1663	0		0	0	0
915	1664	0		0	0	0
916	1665	0		0	0	0
917	1666	0		0	0	0
918	1667	0		0	0	0
919	1668	0		0	0	0
920	1669	0		0	0	0
921	1670	0		0	0	0
922	1671	0		0	0	0
923	1672	0		0	0	0
924	1673	0		0	0	0
925	1674	0		0	0	0
926	1675	0		0	0	0
927	1676	0		0	0	0
928	1677	0		0	0	0
929	1678	0		0	0	0
930	1679	0		0	0	0
931	1680	0		0	0	0
932	1681	0		0	0	0
933	1682	0		0	0	0
934	1683	0		0	0	0
935	1684	0		0	0	0
936	1685	0		0	0	0
937	1686	0		0	0	0
938	1687	0		0	0	0
939	1688	0		0	0	0
940	1689	0		0	0	0
941	1690	0		0	0	0

942	1691	0		0	0	0
943	1692	0		0	0	0
944	1693	0		0	0	0
945	1694	0		0	0	0
946	1695	0		0	0	0
947	1696	0		0	0	0
948	1697	0		0	0	0
949	1698	0		0	0	0
950	1699	0		0	0	0
951	1700	0		0	0	0
952	1701	0		0	0	0
953	1702	0		0	0	0
954	1703	0		0	0	0
955	1704	0		0	0	0
956	1705	0		0	0	0
957	1706	0		0	0	0
958	1707	0		0	0	0
959	1708	0		0	0	0
960	1709	0		0	0	0
961	1710	0		0	0	0
962	1711	0		0	0	0
963	1712	0		0	0	0
964	1713	0		0	0	0
965	1714	0		0	0	0
966	1715	0		0	0	0
967	1716	0		0	0	0
968	1717	0		0	0	0
969	1718	0		0	0	0
970	1719	0		0	0	0
971	1720	0		0	0	0
972	1721	0		0	0	0
973	1722	0		0	0	0
974	1723	0		0	0	0
975	1724	0		0	0	0
976	1725	0		0	0	0
977	1726	0		0	0	0
978	1727	0		0	0	0
979	1728	0		0	0	0
980	1729	0		0	0	0
981	1730	0		0	0	0
982	1731	0		0	0	0
983	1732	0		0	0	0
984	1733	0		0	0	0

985	1734	0		0	0	0
986	1735	0		0	0	0
987	1736	0		0	0	0
988	1737	0		0	0	0
989	1738	0		0	0	0
990	1739	0		0	0	0
991	1740	0		0	0	0
992	1741	0		0	0	0
993	1742	0		0	0	0
994	1743	0		0	0	0
995	1744	0		0	0	0
996	1745	0		0	0	0
997	1746	0		0	0	0
998	1747	0		0	0	0
999	1748	0		0	0	0
1000	1749	0		0	0	0
1001	1750	0		0	0	0
1002	1751	0		0	0	0
1003	1752	0		0	0	0
1004	1753	0		0	0	0
1005	1754	0		0	0	0
1006	1755	0		0	0	0
1007	1756	0		0	0	0
1008	1757	0		0	0	0
1009	1758	0	0	0	0	0
1010	1759	0		0	0	0
1011	1760	0		0	0	0
1012	1761	0		0	0	0
1013	1762	0		0	0	0
1014	1763	0		0	0	0
1015	1764	0		0	0	0
1016	1765	0		0	0	0
1017	1766	0		0	0	0
1018	1767	0		0	0	0
1019	1768	0		0	0	0
1020	1769	0		0	0	0
1021	1770	0		0	0	0
1022	1771	0	0	0	0	0
1023	1772	0		0	0	0
1024	1773	0		0	0	0
1025	1774	0		0	0	0
1026	1775	0	0	0	0	0
1027	1776	0		0	0	0

1028	1777	0		0	0	0
1029	1778	0		0	0	0
1030	1779	0		0	0	0
1031	1780	0		0	0	0
1032	1781	0		0	0	0
1033	1782	0		0	0	0
1034	1783	0		0	0	0
1035	1784	0		0	0	0
1036	1785	0		0	0	0
1037	1786	0		0	0	0
1038	1787	0		0	0	0
1039	1788	0		0	0	0
1040	1789	0		0	0	0
1041	1790	0	0	0	0	0
1042	1791	0		0	0	0
1043	1792	0	0	0	0	0
1044	1793	0	0	0	0	0
1045	1794	0		0	0	0
1046	1795	0		0	0	0
1047	1796	0		0	0	0
1048	1797	0		0	0	0
1049	1798	0		0	0	0
1050	1799	0		0	0	0
1051	1800	0		0	0	0
1052	1801	0	0	0	0	0
1053	1802	0	0	0	0	0
1054	1803	0	0	0	0	0
1055	1804	0		0	0	0
1056	1805	0		0	0	0
1057	1806	0		0	0	0
1058	1807	0		0	0	0
1059	1808	0		0	0	0
1060	1809	0		0	0	0
1061	1810	0		0	0	0
1062	1811	0		0	0	0
1063	1812	0		0	0	0
1064	1813	0		0	0	0
1065	1814	0		0	0	0
1066	1815	0		0	0	0
1067	1816	0		0	0	0
1068	1817	0		0	0	0
1069	1818	0		0	0	0
1070	1819	0		0	0	0

1071	1820	0		0	0	0
1072	1821	0		0	0	0
1073	1822	0	0	0	1	0
1074	1823	0		0	0	0
1075	1824	0	0	0	0	0
1076	1825	0	0	0	0	0
1077	1826	0		0	0	0
1078	1827	0		0	0	0
1079	1828	0	0	0	0	0
1080	1829	0	0	0	0	0
1081	1830	0	0	0	0	0
1082	1831	0	0	0	0	0
1083	1832	0		0	0	0
1084	1833	0	0	0	0	0
1085	1834	0		0	0	0
1086	1835	0	0	0	0	0
1087	1836	0		0	0	0
1088	1837	0		0	0	0
1089	1838	0		0	0	0
1090	1839	0		0	0	0
1091	1840	0	0	0	0	0
1092	1841	0		0	0	0
1093	1842	0		0	0	0
1094	1843	0		0	0	0
1095	1844	0		0	0	0
1096	1845	0		0	0	0
1097	1846	0		0	0	0
1098	1847	0	0	0	0	0
1099	1848	0		0	0	0
1100	1849	0		0	0	0
1101	1850	0		0	0	0
1102	1851	0		0	0	0
1103	1852	0		0	0	0
1104	1853	0		0	0	0
1105	1854	0		0	0	0
1106	1855	0	0	0	0	0
1107	1856	0		0	0	0
1108	1857	0		0	0	0
1109	1858	0		0	0	0
1110	1859	0		0	0	0
1111	1860	0		0	0	0
1112	1861	0		0	0	0
1113	1862	0		0	0	0

1114	1863	0		0	0	0
1115	1864	0		0	0	0
1116	1865	0		0	0	0
1117	1866	0		0	0	0
1118	1867	0	0	0	0	0
1119	1868	0		0	0	0
1120	1869	0		0	0	0
1121	1870	0	0	0	0	0
1122	1871	0		0	0	0
1123	1872	0		0	0	0
1124	1873	0		0	0	0
1125	1874	0		0	0	0
1126	1875	0		0	0	0
1127	1876	0		0	0	0
1128	1877	0		0	0	0
1129	1878	0		0	0	0
1130	1879	0		0	0	0
1131	1880	0		0	0	0
1132	1881	0		0	0	0
1133	1882	0		0	0	0
1134	1883	0		0	0	0
1135	1884	0		0	0	0
1136	1885	0		0	0	0
1137	1886	0		0	0	0
1138	1887	0		0	0	0
1139	1888	0		0	0	0
1140	1889	0		0	0	0
1141	1890	0		0	0	0
1142	1891	0		0	0	0
1143	1892	0		0	0	0
1144	1893	0		0	0	0
1145	1894	0		0	0	0
1146	1895	0		0	0	0
1147	1896	0		0	0	0
1148	1897	0		0	0	0
1149	1898	0		0	0	0
1150	1899	0		0	0	0
1151	1900	0		0	0	0
1152	1901	0		0	0	0
1153	1902	0		0	0	0
1154	1903	0		0	0	0
1155	1904	0		0	0	0
1156	1905	0		0	0	0

1157	1906	0		0	0	0
1158	1907	0		0	0	0
1159	1908	0		0	0	0
1160	1909	0		0	0	0
1161	1910	0		0	0	0
1162	1911	0		0	0	0
1163	1912	0		0	0	0
1164	1913	0		0	0	0
1165	1914	0	0	0	0	0
1166	1915	0		0	0	0
1167	1916	0		0	0	0
1168	1917	0		0	0	0
1169	1918	0		0	0	0
1170	1919	0		0	0	0
1171	1920	0		0	0	0
1172	1921	0		0	0	0
1173	1922	0		0	0	0
1174	1923	0		0	0	0
1175	1924	0		0	0	0
1176	1925	0		0	0	0
1177	1926	0		0	0	0
1178	1927	0		0	0	0
1179	1928	0		0	0	0
1180	1929	0		0	0	0
1181	1930	0		0	0	0
1182	1931	0		0	0	0
1183	1932	0		0	0	0
1184	1933	0		0	0	0
1185	1934	0		0	0	0
1186	1935	0	0	0	0	0
1187	1936	0		0	0	0
1188	1937	0		0	0	0
1189	1938	0		0	0	0
1190	1939	0		0	0	0
1191	1940	0		0	0	0
1192	1941	0		0	0	0
1193	1942	0		0	0	0
1194	1943	0		0	0	0
1195	1944	0		0	0	0
1196	1945	0		0	0	0
1197	1946	0		0	0	0
1198	1947	0		0	0	0
1199	1948	0		0	0	0

1200	1949	0		0	0	0
1201	1950	0		0	0	0
1202	1951	0		0	0	0
1203	1952	0		0	0	0
1204	1953	0		0	0	0
1205	1954	0		0	0	0
1206	1955	0		0	0	0
1207	1956	0		0	0	0
1208	1957	0		0	0	0
1209	1958	0		0	0	0
1210	1959	0		0	0	0
1211	1960	0		0	0	0
1212	1961	0		0	0	0
1213	1962	0		0	0	0
1214	1963	0	0	0	0	0
1215	1964	0		0	0	0
1216	1965	0		0	0	0
1217	1966	0		0	0	0
1218	1967	0		0	0	0
1219	1968	0		0	0	0
1220	1969	0		0	0	0
1221	1970	0		0	0	0
1222	1971	0		0	0	0
1223	1972	0		0	0	0
1224	1973	0		0	0	0
1225	1974	0		0	0	0
1226	1975	0		0	0	0
1227	1976	0		0	0	0
1228	1977	0		0	0	0
1229	1978	0		0	0	0
1230	1979	0		0	0	0
1231	1980	0		0	0	0
1232	1981	0		0	0	0
1233	1982	0		0	0	0
1234	1983	0		0	0	0
1235	1984	0		0	0	0
1236	1985	0		0	0	0
1237	1986	0		0	0	0
1238	1987	0		0	0	0
1239	1988	0		0	0	0
1240	1989	0		0	0	0
1241	1990	0		0	0	0
1242	1991	0	0	0	0	0

1243	1992	0		0	0	0	0
1244	1993	0		0	0	0	0
1245	1994	0		0	0	0	0
1246	1995	0	0	0	0	0	0
1247	1996	0	0	0	1	0	0
1248	1997	0	0	0	0	0	0
1249	1998	0	0	0	0	0	0
1250	1999	0	0	0	0	0	0
1251	2000	0	0	0	0	0	0
1252	2001	0	0	0	0	0	0
1253	2002	0	0	0	0	0	0
1254	2003	0	0	0	0	0	0
1255	2004	0	0	0	0	0	0
1256	2005	0	0	0	0	0	0
1257	2006	0	0	0	0	0	0
1258	2007	0	0	0	0	0	0
1259	2008	0	0	0	0	0	0
1260	2009	0	0	0	0	0	0
1261	2010	0	0	0	0	0	0
1262	2011	0	0	0	0	0	0
1263	2012	0	0	0	1	0	0
1264	2013	0	0	0	0	0	0
1265	2014	0	0	0	0	0	0
